# A comparison of multiple amyloid PET radiotracers for Down syndrome clinical trials

**DOI:** 10.1093/braincomms/fcaf406

**Published:** 2025-10-15

**Authors:** Matthew Zammit, Julie Price, Bradley Christian, Michael Rafii, the Alzheimer’s Biomarker Consortium-Down Syndrome, Beau M Ances, Howard F Andrews, Karen Bell, Rasmus M Birn, Adam M Brickman, Peter Bulova, Jeff Burns, Amrita Cheema, Kewei Chen, Bradley T Christian, Isabel Clare, Ann D Cohen, Eric W Doran, Tatiana M Foroud, Benjamin L Handen, Jordan Harp, Sigan L Hartley, Elizabeth Head, Denise Head, Christy Hom, Lawrence Honig, Milos D Ikonomovic, Sterling C Johnson, M Ilyas Kamboh, David Keator, Julia K Kofler, William Charles Kreisl, Sharon J Krinsky-McHale, Florence Lai, Patrick Lao, Charles Laymon, Joseph Hyungwoo Lee, Ira T Lott, Victoria Lupson, Mark Mapstone, Davneet S Minhas, Neelesh Nadkarni, Sid O’Bryant, Deborah Pang, Melissa Petersen, Julie C Price, Lauren Ptomey, Margaret Pulsifer, Michael S Rafii, Herminia Diana Rosas, Frederick Schmitt, Nicole Schupf, Wayne P Silverman, Dana L Tudorascu, Rameshwari Tumuluru, Badri Varadarajan, Michael A Yassa, Shahid Zaman, Fan Zhang

**Affiliations:** University of Wisconsin-Madison, Waisman Center, Madison, WI 53705, USA; Massachusetts General Hospital/Harvard Medical School, Department of Radiology, Boston, MA 02114, USA; University of Wisconsin-Madison, Waisman Center, Madison, WI 53705, USA; Alzheimer’s Therapeutic Research Institute, Keck School of Medicine of University of Southern California, San Diego, CA 92121, USA

**Keywords:** Down syndrome, Alzheimer's disease, amyloid PET, clinical trials

## Abstract

Adults with Down syndrome carry high risk of developing Alzheimer’s disease and efforts to include this population in clinical trials remain limited. A barrier to recruitment for anti-amyloid trials includes the availability of the same amyloid PET radiotracer to multiple treatment centres. The objective of the study is to compare longitudinal rates of change between different amyloid PET radiotracers, particularly Pittsburgh compound B and florbetapir, in Down syndrome and to compare the estimated age at amyloid-positivity derived from these radiotracers. Two hundred thirty-seven adults with Down syndrome from the Trial Ready Cohort-Down syndrome and Alzheimer’s Biomarker Consortium-Down syndrome studies were imaged using T_1_-weighted MRI and using PET images of Pittsburgh compound B, florbetapir, NAV4694 or flutemetamol to screen for amyloid plaque burden. Currently, Pittsburgh compound B and florbetapir have longitudinal data from these cohorts, while NAV4694 has one individual with longitudinal scans and flutemetamol has no available longitudinal data. Pittsburgh compound B displayed a greater effect size to measure amyloid change compared to florbetapir. NAV4694 and Pittsburgh compound B, which are structurally similar compounds, displayed similar sensitivity to measure longitudinal amyloid increase. The estimated age at amyloid onset showed no significant difference between Pittsburgh compound B, florbetapir, NAV4694 or flutemetamol. The findings suggest that different amyloid PET radiotracers provide consistent estimates of amyloid onset age for adults with Down syndrome. Multicentre studies of Alzheimer’s disease therapeutics can utilize multiple amyloid PET radiotracers to facilitate recruitment; however, these radiotracers have different sensitivity to detect longitudinal change.

## Teaser

Zammit *et al*. report that participants with Down syndrome imaged with Pittsburgh compound B, florbetapir, NAV4694 or flutemetamol show no difference in age at amyloid onset despite their different sensitivities to detect amyloid change. They conclude that Alzheimer’s disease clinical trials can utilize multiple amyloid imaging agents to facilitate recruitment.

## Graphical Abstract

**Graphical Abstract:**
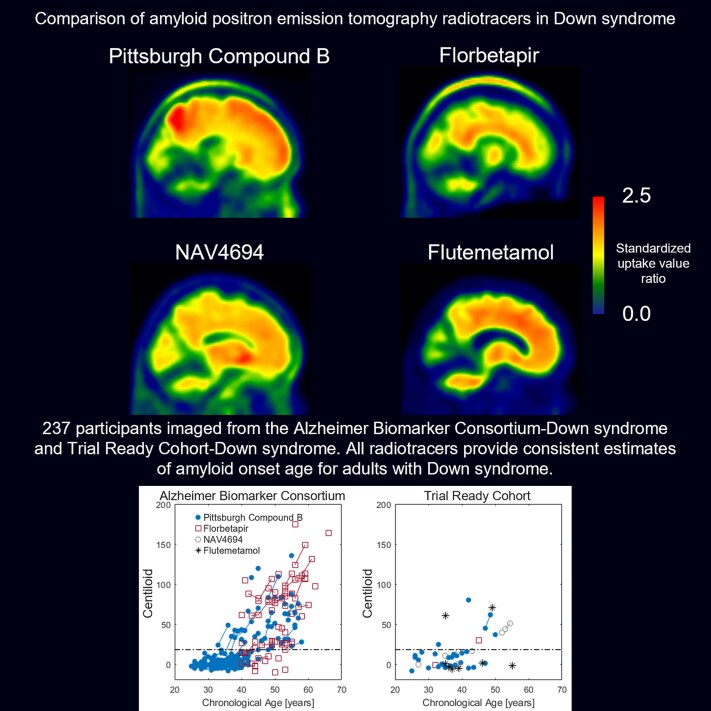

## Introduction

Individuals with Down syndrome (DS) represent the largest population of genetically determined Alzheimer’s disease (AD).^1^ This genetic form of AD is driven by triplication of chromosome 21, which encodes the gene responsible for amyloid precursor protein production, leading to beta-amyloid (Aβ) plaque deposition early in life. AD is accountable for 70% of deaths in this demographic over the age of 35.^1-3^ The prevalence of AD dementia sharply increases past 50 years of age and the average age of dementia onset is 55 years.^4^ As the lifetime risk of AD in DS exceeds 90%,^5,6^ DS may serve as a model population for understanding both the progression of the disease and the long-term benefits of early therapeutic interventions. Clinical trials in neurotypical populations during early-stage dementia have shown the success of anti-amyloid therapies at removing Aβ and slowing the progression of clinical symptoms.^7^ AD biomarker studies suggest these drugs may be more effective for secondary prevention by administering during the ‘preclinical’ timeframe before clinical symptoms emerge. Despite the severity of AD risk in DS, this population has not been included in recent anti-amyloid clinical trials, resulting from factors such as limited access to expert clinical evaluation for dementia, challenges in assessing cognitive change in the setting of intellectual disability, concerns regarding cerebral amyloid angiopathy and the presumed increased risk of adverse events related to amyloid-related imaging abnormalities common with these therapies.^1,8^

Amyloid plaque pathology can be detected with PET imaging and amyloid burden increases with age.^9^ Anti-amyloid therapeutic trials utilize PET outcome measures to confirm target engagement and rely on radiotracers labelled with ^18^F that allow for regional distribution to treatment centres. Many large DS imaging studies have historically used ^11^C labelled Pittsburgh compound B (PiB), which cannot be distributed in a multicentre trial due to its short half-life, and a knowledge gap exists on how ^18^F radiotracers evaluate longitudinal Aβ change with respect to AD staging in this population. To address these limitations and to improve the feasibility of DS recruitment for clinical trials, we present the first multi-radiotracer comparison for longitudinal Aβ change from the Trial Ready Cohort-Down syndrome (TRC-DS) and the Alzheimer’s Biomarker Consortium-Down syndrome (ABC-DS). The objective of the study is to compare longitudinal rates of change between different amyloid PET radiotracers, particularly PiB and florbetapir (FBP), in DS and to compare the estimated age at amyloid-positivity derived from these radiotracers.

## Materials and methods

A total of 237 adults with DS were recruited from the TRC-DS and ABC-DS studies. Participants from each cohort were included in the study if they had available and quality checked MRI and amyloid PET. Institutional Review Board approval and informed consent were obtained during enrolment into the study by the participant or legally designated caregiver according to the Declaration of Helsinki. Participant demographics are provided in Table 1. All participants underwent T_1_-w MRI imaging, as well as PET imaging using either PiB, FBP, NAV4694 (NAV) or flutemetamol (FMM) across multiple study visits (Table 1). Omnibus comparisons between participants categorized by radiotracer were performed using chi-squared tests and ANOVA. Statistical significance in the demographics were primarily driven by the older age of the cohort imaged with FBP. For longitudinal imaging, 76 participants were imaged with PiB, 22 were imaged with FBP and 1 participant was imaged with NAV. All participants with longitudinal imaging received the same radiotracer for each visit. While NAV has only one participant with longitudinal data and FMM currently has only cross-sectional data, these data remained in the analyses for cross-sectional comparisons between all radiotracers. Imaging protocols were standard across every imaging site. For MRI, T_1_-w scans were acquired using a sagittal 3D accelerated MPRAGE/IRSPGR sequence. Targeted injected activity of 15 mCi PiB, 8 mCi NAV, 10 mCi FBP and 5 mCi FMM were administered to the participants. Scans were acquired for 20 min and began 50 min post-injection for PiB, NAV and FBP while FMM scans began 90 min post-injection. Global Aβ burden was determined using the standard SPM8 Centiloid (CL) method^10^ using the whole cerebellum as the reference tissue for PiB, NAV and FMM, while both the cerebellum and an eroded white matter reference were used for FBP. The eroded white matter reference regions were generated for each individual from segmentation maps of the T_1_-w MRI created from the SPM8 normalization procedure described below. These maps were thresholded (0.9) and used to sample the average PET standardized uptake value for whole brain white matter for each individual. Briefly, PET images were initially co-registered with the T_1_-w MRI, the MRI was then spatially normalized to MNI152 template space and the PET images were subsequently transformed to template space using the MRI normalization parameters. PET images were then smoothed using a Gaussian smoothing kernel to a target point spread function of 8 mm to account for PET scanner resolution differences across imaging sites. Standardized uptake value ratio (SUVR) images were then generated by voxel normalization to the mean activity in the reference tissue, and the mean cortical SUVR was extracted from the Centiloid cortex region of interest (ROI). Cortical SUVR was then converted to units of CL using the appropriate conversion equations for PiB,^10^ FBP,^11^ NAV^12^ and FMM.^13^ In addition, SUVR values were extracted from a striatum ROI to assess the extent of early striatal Aβ in these participants as previously observed with PiB.^14,15^ Participants were classified as amyloid-positive (A+) using an a priori threshold of 18 CL.^16,17^ The threshold of 18 CL corresponds to an SUVR of 1.20 for PiB, 1.14 for FBP, 1.24 for NAV and 1.15 for FMM. The 18 CL threshold was previously derived from a sample of participants with DS imaged with PiB that showed very early signs of longitudinal Aβ increase, and was found as an optimal threshold that had the highest area under the curve (AUC) (0.91) from receiver operating characteristic (ROC) analysis compared to more traditional CL thresholds (e.g. 24–30 CL) as well as lower CL thresholds (e.g. 12 CL).^17^ Using a sampled iterative local approximation (SILA) algorithm,^18^ a longitudinal CL trajectory model was created for the DS population as described previously from the ABC-DS.^16,19^ Briefly, the participant’s PET CL outcomes were fit to the model to determine their ‘amyloid age’, or the estimated number of years since becoming PET A+. amyloid age was then subtracted from the participants chronological age at their last scan to determine their estimated age at amyloid onset. The SILA algorithm offers a unique way to evaluate differences in rates of change between radiotracers against a population-averaged amyloid timeline. SILA was designed to effectively incorporate both cross-sectional and longitudinal datasets to compare across the same population-averaged trajectory such that participants with only a single timepoint can receive an amyloid age estimate.^18^

**Table 1:** Participant demographics from ABC-DS and TRC-DS categorized by radiotracer

	PiB	FBP	NAV	FMM	Omnibus comparison (*P*)
Participants (*N*)	155	66	7	9	—
Sex (*N* female [%])	70 [45%]	23 [35%]	3 [43%]	3 [33%]	0.5
Baseline age (years [SD])	38.6 [8.4]	49.9 [6.4]	42.8 [9.1]	42.0 [6.8]	0.00001
Participants with longitudinal scans (%)	76 [49%]	22 [33%]	1 [14%]	0 [0%]	0.0002
Baseline CL (mean [SD])	17.8 [28.2]	55.3 [44.3]	17.7 [16.3]	13.0 [28.3]	0.00001
*N* cognitively impaired [%]	17 [11%]	15 [23%]	1 [14%]	0 [0%]	0.001

Statistical comparisons performed using chi-squared tests and one-way ANOVA. Cognitive impairment is defined as having either mild cognitive impairment or dementia as determined through a cognitive consensus.

### Statistical analyses

Rates of longitudinal Aβ change were measured in CL/year for each radiotracer, and Cohen’s *d* effect sizes^20^ were calculated to evaluate the differences in the annualized rate of change between the baseline and most recent follow-up measure (i.e. the measure with the longest delay from baseline). Estimated amyloid onset ages were then compared between the radiotracers using one-way ANOVA with Tukey’s honestly significant difference (HSD) for multiple comparisons. An alpha of *P* < 0.05 was used to determine statistical significance. For a better understanding of the cross-sectional-only cohorts for NAV and FMM, we performed ANOVA between baseline age and CL in the TRC-DS cohort.

## Results

**Figure 1:**
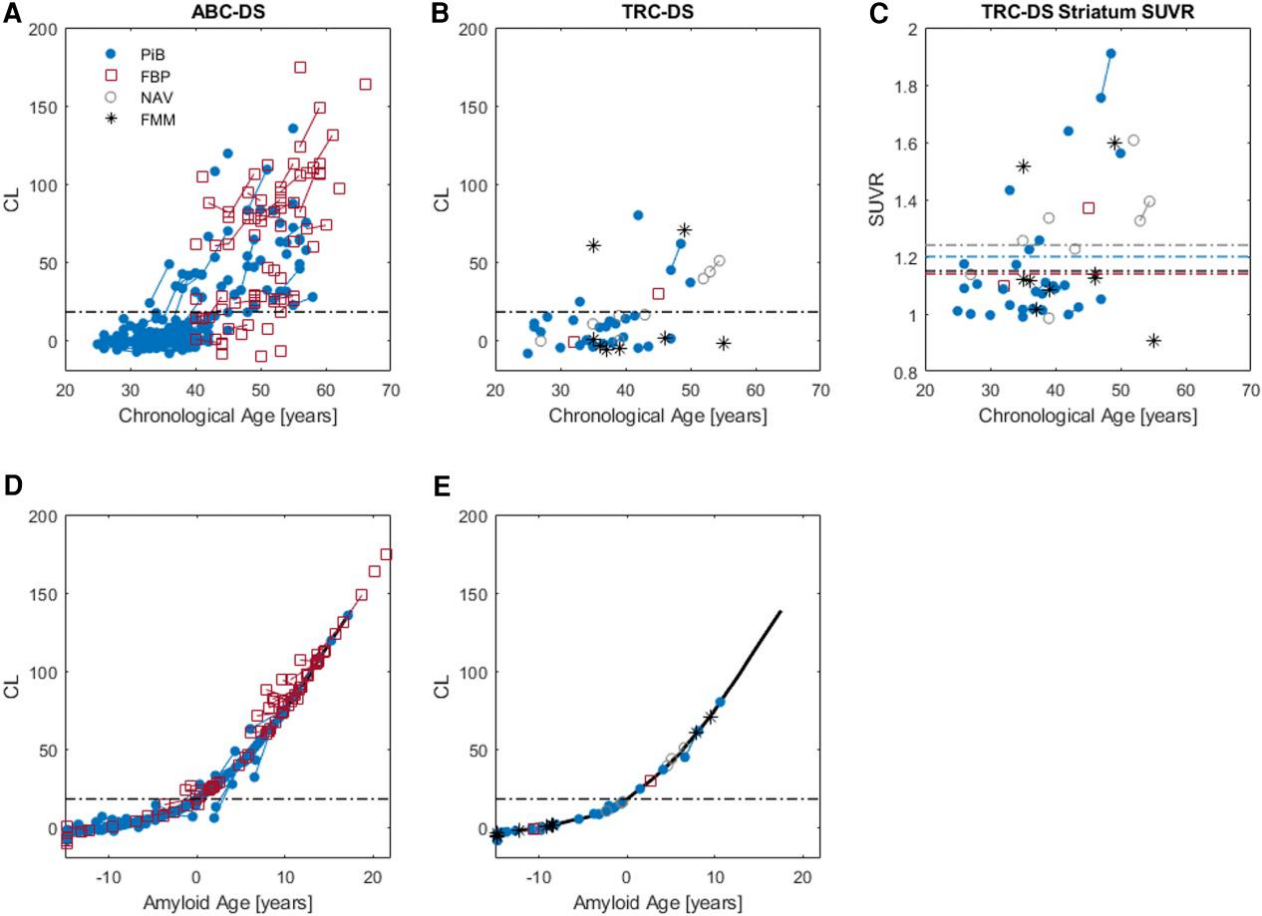
**Cross-sectional (single point) and longitudinal (points connected by lines) amyloid trajectories for the *N* = 237 participants from the ABC-DS and the TRC-DS.** (**A**) Amyloid PET CL with respect to chronological age for ABC-DS participants evaluated with PiB and FBP. (**B**) Amyloid PET CL with respect to chronological age for TRC-DS participants evaluated with PiB, FBP, NAV and FMM. (**C**) Striatum SUVRs with respect to age for PiB, FBP, NAV and FMM. (**D**) Modelled longitudinal trajectories displaying CL with respect to amyloid age in ABC-DS. (**E**) Modelled longitudinal trajectories displaying CL with respect to amyloid age in TRC-DS. Dashed lines represent the a priori threshold for amyloid positivity (A+) of 18 CL. The solid black curve in **D** and **E** represents the previously established CL versus amyloid age trajectory for the DS population. Dashed lines represent the cutoffs for A+. CL values were compared across each radiotracer using ANOVA with Tukey’s honestly significant difference (HSD) test for multiple comparisons. ANOVA revealed significant differences between radiotracer binding (*P* = 0.00001). Tukey’s HSD revealed that FBP binding was significantly higher (*P* < 0.05) than all other radiotracers due to the older age of the cohort imaged with FBP and that no significant difference was observed in binding between PiB, NAV and FMM (*P* > 0.05).

**Figure 2:**
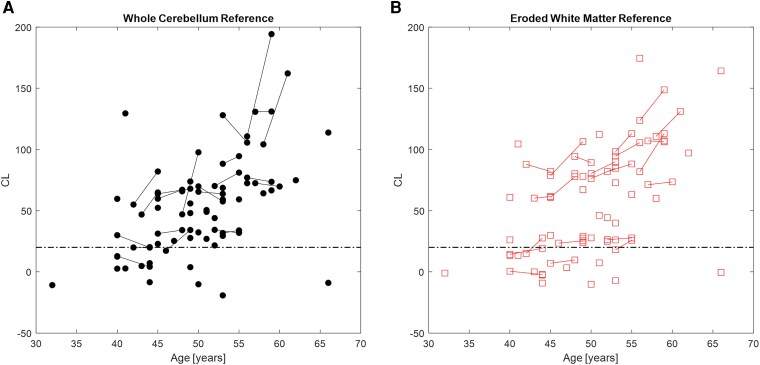
**Cross-sectional and longitudinal FBP trajectories for the *N* = 66 participants from the ABC-DS and the TRC-DS determined using a whole cerebellum reference region (left) and an eroded white matter reference region (right).** (**A**) FBP CL obtained using a whole cerebellum reference region. (**B**) FBP CL obtained using an eroded white matter reference region. Single points represent cross-sectional FBP CL for a given participant, while points connected by lines represent participants with longitudinal FBP measures. The dashed lines represent the cutoff for amyloid positivity (A+). Baseline CL values for the FBP data obtained from the cerebellum (left) and eroded white matter (right) reference regions were compared using an ANOVA *F* test. No significant differences were observed between CL values computed using the different reference regions (*P* > 0.05).

Amyloid PET outcomes using PiB, FBP and NAV displayed longitudinal increases with respect to both chronological age and amyloid age in the TRC-DS and ABC-DS cohorts (Fig. 1). Longitudinal FMM data were not obtained during this study. Table 2 provides longitudinal outcomes for each radiotracer as well as the estimated amyloid onset ages determined for these cohorts. For participants imaged with PiB, the mean baseline amyloid burden in the A+ group was 40 [18] CL while the follow-up amyloid burden was 58 [22] CL, corresponding to an effect size (Cohen’s *d*) of 0.9. Participants scanned with FBP using the white matter reference displayed mean baseline amyloid burden of 70 [33] CL, and follow-up burden of 80 [37] CL, corresponding to an effect size of 0.3. For FBP with the whole cerebellum reference region, the mean baseline amyloid burden was 67 [32] CL and the follow-up burden was 76 [44] CL, corresponding to an effect size of 0.2. Figure 2 displays the cross-sectional and longitudinal FBP trajectories for participants from both ABC-DS and TRC-DS comparing CLs obtained with the whole cerebellum and white matter reference tissues. Only one participant received longitudinal NAV imaging, and this individual had a baseline and follow-up amyloid burden of 44 and 51 CL, respectively. The mean baseline amyloid burden for all participants scanned with NAV was 41.5 [2.2] CL, while the mean baseline burden for FMM was 65.5 [5.2] CL. Longitudinal rates of amyloid change were calculated as 7.3 [4.1], 3.3 [3.5] and 4.9 [0.0] CL/year for PiB, FBP and NAV, respectively. From ANOVA, participants scanned with PiB displayed significantly higher rates of amyloid increase compared to FBP [*F*(df) = 8.2(1); *P* = 0.007]. Following temporal modelling to align the participants’ trajectories to the population-averaged amyloid age curve, the estimated age at A+ onset was determined as 41.9 [6.4] years for PiB, 42.9 [6.0] years for FBP, 43.2 [4.7] years for NAV and 47.0 [9.1] years for FMM. From ANOVA, no significant differences in estimated age at A+ onset were observed between radiotracers [*F*(df) = 1.4(1); *P* = 0.23]. Multiple comparison analysis with Tukey’s HSD revealed no significant differences in estimated age at A+ between each radiotracer. For striatal Aβ, all participants that were A+ in the cortex were also A+ in the striatum across PiB, FBP, NAV and FMM. In the TRC-DS cohort, two participants imaged with PiB and two participants imaged with NAV were A+ in the striatum but A− in the cortex. To evaluate potential radiotracer differences with the cross-sectional-only data, ANOVA was performed on baseline age and CL values in the TRC-DS cohort. The mean CL (SD) for each radiotracer is as follows: PiB = 10.7 (20.1) CL, FBP = 14.4 (15.4) CL, NAV = 17.7 (16.3) CL, FMM = 13.0 (28.3) CL. ANOVA revealed no significant differences between baseline CL across the radiotracers [*F*(df) = 0.25(3), *P* = 0.78]. The mean age (SD) for each participant imaged with each radiotracer is as follows: PiB = 35.5 (7.0) years, FBP = 38.5 (6.5) years, NAV = 41.1 (8.5) years, FMM = 42.0 (6.8) years. ANOVA revealed no significant differences between baseline age of participants across the radiotracers [*F*(df) = 3.1 (3), *P* = 0.60].

**Table 2:** Amyloid onset age, amyloid PET burden, the rate of longitudinal amyloid increase and Cohen’s *d* effect sizes for participants with DS scanned with PiB, FBP, NAV and FMM

PET radiotracer	Estimated amyloid onset age (years [SD])	A+ baseline amyloid burden (CL [SD])	A+ follow-up amyloid burden (CL [SD])	A+ longitudinal rate of change (CL/year [SD])	Cohen’s *d* effect size
PiB	41.9 [6.4]	40 [18]	58 [22]	7.3 [4.1]	0.9
FBP (eroded white matter reference)	42.9 [6.0]	70 [33]	80 [37]	3.3 [3.5]	0.3
FBP (whole cerebellum reference)	45.2 [6.5]	67 [32]	76 [44]	4.0 [8.9]	0.2
NAV	43.2 [4.7]	41.5 [2.2]	51 [0.0]^a^	4.9 [0.0]^a^	N/A^a^
FMM	47.0 [9.1]	65.5 [5.2]	N/A^b^	N/A^b^	N/A^b^

Amyloid burden is presented in units of CL.

^a^Only one participant had longitudinal NAV scans.

^b^No longitudinal FMM scans were performed during this study.

## Discussion

The low prevalence of DS (∼12.6 in 10 000 births) in the USA^21^ poses a challenge for clinical trial recruitment, especially within the age range where AD-related change begins. This study represents the first multi-radiotracer comparison of amyloid change for the DS population to facilitate DS trial recruitment. This study also represents a large, multicentre project to collect AD biomarker data and stage the DS population for disease-modifying clinical trials. Historically, ABC-DS published amyloid imaging findings using PiB PET, and this new data provides comparisons of PiB PET to FBP, NAV and FMM with the addition of the TRC-DS dataset. When evaluating the amyloid PET findings at the cross-sectional level, all four radiotracers were able to provide similar estimates in the age at amyloid onset. This suggests that use of any amyloid PET radiotracer should be suitable for the purposes of staging an individual within the AD continuum and for the purposes of clinical trial screening. For longitudinal analysis, PiB, FBP and NAV were able to detect longitudinal amyloid increase between baseline and follow-up measures, with TRC-DS having the shortest follow-up of ∼1.5 years, which aligns with the timeline of anti-amyloid trial endpoints. Longitudinal imaging data for FMM as part of TRC-DS was not available for the current study. Longitudinal FMM data will be collected as the TRC-DS study progresses. PiB displayed a greater effect size to detect longitudinal amyloid increase compared to FBP, which displayed a smaller effect size, likely a result of its lower signal dynamic range.^11^ In the current study, we chose to use an eroded white matter reference region for FBP as studies suggest improved quantitative accuracy over the cerebellum.^22^ However, the sample of adults scanned with FBP were in an age range where cognitive impairment is more prevalent, yet the sensitivity of FBP to detect Aβ change was much lower than the sensitivity of PiB measured in a younger cohort, suggesting poor longitudinal performance of FBP. This effect could likely be a result of slower Aβ progression during the later disease stage in the older adults with DS as the rate of Aβ accumulation slows. For a more direct comparison of FBP to PiB, NAV and FMM, we repeated the analysis of FBP images using the whole cerebellum as the reference tissue. With a whole cerebellum reference tissue, the sensitivity of FBP to detect longitudinal increase in amyloid decreased (Cohen’s *d*: 0.2) when compared to the white matter reference region (Cohen’s *d*: 0.3). This concern is further illustrated by the observance of seven participants of A+ status that revealed a decrease in Aβ burden at follow-up with the whole cerebellum reference region, compared to only two participants showing a decrease with the white matter reference for FBP, and zero A+ participants imaged with PiB. Studies have shown that FBP does not correlate well with PiB following CL conversion, and a potential source of variability can be linked to partial volume effects.^23^ With partial volume correction, variability of FBP can be reduced while improving the correlation with PiB, albeit this has shown to increase the variance with FBP.^23^ As more data is collected, the influence of partial volume correction on CL outcomes with different amyloid PET radiotracers can be more accurately assessed in the DS population. While limited in sample size, NAV displayed greater longitudinal increase compared to FBP. NAV results could be anticipated to be closely aligned with PiB, as binding and *in vivo* kinetics have been shown to be highly correlated with PiB while displaying lower off-target white matter signal^24^ compared to FBP. NAV and PiB are structurally similar,^12^ resulting in similar *in vivo* performance. For the striatal Aβ data, PiB, FBP, NAV and FMM all displayed significant striatal binding with increased cortical Aβ burden. All participants that were cortically A+ were also A+ in the striatum. For both PiB and NAV, there were four participants in total from TRC-DS that displayed striatal A+ while remaining cortically A−. This result further highlights previous findings that initial build-up of diffuse Aβ plaques in the striatum^25^ can be detected early with PET in DS^14,15^ and that these striatal changes can identify individuals suitable for anti-amyloid therapies 3–4 years earlier compared to using cortical Aβ alone as the outcome.^15^ For a better understanding of amyloid binding in our cross-sectional-only datasets, additional ANOVA analyses were performed for the TRC-DS cohort, which is both age and sex-matched. At baseline, participants imaged with PiB, FBP, NAV or FMM displayed no differences in age or CL burden, highlighting the similarities between each radiotracer at detecting Aβ plaques in similar individuals.

Previous AD-related studies in the DS population highlight the heterogeneity in the age at amyloid onset.^17,26^ Thus, clinical trial recruitment based upon age alone would result in significantly high screening fail rates to achieve a suitable sample size. A recent study observing the prevalence of Aβ positivity identified that only 5% of individuals between the ages of 35 and 39 were A+^9^ This prevalence increased to ∼57% between the ages of 40 and 44. In DS it has been observed that both neurofibrillary tau deposition^16^ and cognitive decline^27,28^ occur rapidly following the onset of amyloid, with some estimates showing change as early as 5 years after PET A+ is reached.^19^ The rapid onset of tau and cognitive decline suggests that a narrow window of opportunity exists to treat AD in DS, highlighting that age-based clinical trial recruitment alone is not sufficient. Use of biomarker data as part of the screening process can help facilitate recruitment by minimizing screening fail rates. In this study, we incorporated use of a SILA algorithm^18^ to align the PET outcomes of our participants to a Alzheimer's disease in DS (DSAD)-specific timeline of amyloid accumulation. This methodology has been performed previously in DS,^19^ allowing for a robust characterization of the progression of AD following Aβ onset. This amyloid clock^29^ framework has the potential to aid clinical trial design for DS as the AD therapeutic field moves towards implementing combination therapies targeting multiple AD pathologies. In the current study, we highlight that individuals with DS can be evaluated within the amyloid clock using multiple amyloid PET radiotracers that display similar estimates in the age at amyloid onset. The estimates for each radiotracer all fall near the median age at A+ onset in DS.^9^ While each radiotracer provided a similar estimate in the age at onset, there is also heterogeneity in the age at amyloid onset in the DS population that spans 25+ years.^16^ The factors influencing this heterogeneity are not well understood. In late-onset AD, factors such as APOE4 carrier status and sex can significantly influence age at amyloid onset, however these factors have not significantly influenced the disease trajectory in DS.^30,31^ This heterogeneity in age at onset can confound age-based predictions of time to symptom onset. Our previous work using the SILA algorithm in DS has shown that use of the amyloid age metric significantly minimizes the variance observed with chronological age-based estimates.^16^ One limitation to this work is that most longitudinal scans were acquired using PiB and FBP, while NAV had a single longitudinal scan and FMM was restricted to cross-sectional analyses. As a result, the SILA estimation for the population-averaged trajectory was primarily driven by PiB and FBP. As longitudinal data for NAV and FMM are collected during the progression of the TRC-DS study, the impact of these data on the SILA trajectory can be evaluated further. Taken together, use of multiple amyloid PET radiotracers can help facilitate clinical trial recruitment in this at-risk population.

The findings suggest that the amyloid PET radiotracers PiB, FBP, NAV and FMM provide consistent estimates of amyloid onset age for adults with DS. Multicentre studies of AD therapeutics can utilize multiple amyloid PET radiotracers to facilitate recruitment; however, these radiotracers have different sensitivity to detect longitudinal Aβ change.

## Data Availability

The imaging sites have entered web-based data through the Alzheimer’s Therapeutic Research Institute (ATRI) as part of the ABC-DS study. The data that support the findings of this study are openly available in the ABC-DS database (https://ida.loni.usc.edu/login.jsp?project=ABCDS). Code for running the SILA algorithm is available at https://github.com/Betthauser-Neuro-Lab/SILA-AD-Biomarker.

## Appendix

Alzheimer's Biomarker Consortium-Down Syndrome (ABC-DS) Investigators:

Beau M. Ances, Howard F. Andrews, Karen Bell, Rasmus M. Birn, Adam M. Brickman, Peter Bulova, Jeff Burns, Amrita Cheema, Kewei Chen, Bradley T. Christian, Isabel Clare, Ann D. Cohen, Eric W. Doran, Tatiana M. Foroud, Benjamin L. Handen, Jordan Harp, Sigan L. Hartley, Elizabeth Head, Denise Head, Christy Hom, Lawrence Honig, Milos D. Ikonomovic, Sterling C Johnson, M. Ilyas Kamboh, David Keator, Julia K. Kofler, William Charles Kreisl, Sharon J. Krinsky-McHale, Florence Lai, Patrick Lao, Charles Laymon, Joseph Hyungwoo Lee, Ira T. Lott, Victoria Lupson, Mark Mapstone, Davneet S. Minhas, Neelesh Nadkarni, Sid O’Bryant, Deborah Pang, Melissa Petersen, Julie C. Price, Lauren Ptomey, Margaret Pulsifer, Michael S. Rafii, Herminia Diana Rosas, Frederick Schmitt, Nicole Schupf, Wayne P. Silverman, Dana L. Tudorascu, Rameshwari Tumuluru, Badri Varadarajan, Michael A. Yassa, Shahid Zaman and Fan Zhang.
